# The Number of Positive Tumor Markers (NPTM) Achieves Higher Value in the Prognosis Prediction of Gastric Cancer

**DOI:** 10.1155/2022/5145918

**Published:** 2022-11-28

**Authors:** Limin Li, Bing Ma, Fubin Liu, Chao Sheng, Yu Peng, Yating Qiao, Peng Wang, Kexin Chen, Fangfang Song

**Affiliations:** Department of Epidemiology and Biostatistics, Key Laboratory of Molecular Cancer Epidemiology, Tianjin, Key Laboratory of Cancer Prevention and Therapy, Tianjin, Key Laboratory of Breast Cancer Prevention and Therapy, Ministry of Education, National Clinical Research Center for Cancer, Tianjin's Clinical Research Center for Cancer, Tianjin Medical University Cancer Institute and Hospital, Tianjin 300060, China

## Abstract

**Purpose:**

The clinical application of combined tumor markers is still limited. We aim to explore the value of the combination of multiple tumor markers in gastric cancer (GC) prognosis.

**Methods:**

The prognostic significance was evaluated using Kaplan–Meier log-rank survival analysis and multivariable Cox regression analysis. The estimated area under the curve (AUC) was compared to evaluate the discriminatory ability of different indicators. A nomogram was constructed based on the results of multivariable cox regression, and its performance was evaluated by Harrell's concordance index and calibration curve.

**Results:**

NPTM (number of positive tumor markers) displayed independent prognostic significance whether in the whole cohort or in patients with different stages. Patients with the all-negative tumor markers had a worse prognosis after postoperative chemotherapy in all cohort (*P* = 0.023) or in age ≤60 subgroup (*P* = 0.012), while patients with positive tumor markers had a better prognosis after postoperative chemotherapy in stage III (*P* = 0.012). The AUC value of NPTM is higher than any individual tumor marker. The 1-, 3-, and 5-year AUC values of the CTNM (combination of NPTM and pTNM) increased by 5%, 4.8%, and 3.6%, respectively, compared with TNM staging system. The nomogram constructed including NPTM showed its high accuracy (C − index = 0.706) versus TNM staging system (C − index = 0.646) and CTNM (C − index = 0.681).

**Conclusions:**

NPTM was an independent predictor of gastric cancer prognosis, showing more accurate prognostic performance than individual tumor markers. Especially its significance in guiding postoperative adjuvant chemotherapy regimens and predicting prognosis by combination with TNM staging system may have a better clinical application value.

## 1. Introduction

Gastric cancer (GC) is one of the common malignant tumors of the digestive tract, and its morbidity and mortality rank fifth and third in global malignancies, respectively [1]. About 1 million GC cases are newly diagnosed every year recently [2]. Moreover, the prognosis of GC is poor. In China, the five-year survival rate of GC patients is less than 50%, which is lower than that of South Korea and Japan in East Asia [3]. Nowadays, the prognosis of GC is mainly predicted by the tumor-node-metastasis (TNM) staging system proposed by the American Joint Committee on Cancer (AJCC) [4]. However, the survival of GC patients at the same disease stage might be heterogeneous [5]. Many other factors such as age, gender, tumor size, tumor location, inflammatory factors, and tumor markers not involved in AJCC staging system might affect the outcomes of GC as well [6–10]. Thus, by integrating these important factors, the individual prognosis of GC patients could be better assessed.

The detection of preoperative serum tumor markers such as CEA, CA19-9, CA24-2, and CA72-4 has been routinely used in a variety of tumor patients. Accumulated data show that these markers are convenient instruments for monitoring recurrence and distant metastasis as well as for evaluating the efficacy of chemotherapy and the prognosis in GC patients [11–13]. However, individual tumor marker's value always has lower sensitivity and specificity and can be easily affected by noncancerous conditions when used for prognostic assessment [14]. Studies [15–17] have shown that the combination with several of the aforementioned tumor markers has higher predictive power for the prognosis of lung, colorectal, and GC. However, few studies have evaluated the prognostic value of tumor markers at different stages of GC and not all tumor markers commonly used are considered.

The purpose of this study was to evaluate the predictive value of the aforementioned four tumor markers and their combination in the prognosis of GC and to verify whether its combination with TNM staging system had a more accurate predictive power for the prognosis of GC.

## 2. Methods

### 2.1. Study Population

Stage I to III GC patients who consecutively underwent curative resection from January 2012 to June 2014 at Tianjin Medical University Cancer Institute and Hospital were enrolled in this study. Our inclusion criteria were as follows: (1) newly-diagnosed and histologically-confirmed adenocarcinoma of the stomach; (2) no evidence of distant metastases; (3) having gastric resections with a curative R0 resection; (4) complete clinic-pathological and follow-up data. The exclusion criteria were as follows: (1) metastatic disease; (2) neoadjuvant chemotherapy or radiotherapy before radical resection of GC; (3) malignant disease of other organs; (4) serious diseases of other organs such as liver cirrhosis and renal failure. Finally, 928 patients were included in this study, and the clinic-pathological characteristics (histological grade, tumor location, tumor size, and pTNM stage according to the eighth corresponding edition of the AJCC Staging Manual) of all patients were abstracted from our hospital information system. The Ethics Committee of Tianjin Medical University Cancer Institute and Hospital approved the study protocol, and written informed consent was obtained from all patients.

We also collected demographic and epidemiologic data for the following analyses. Demographic and epidemiologic factors included age at diagnosis, gender, body mass index (BMI), history of hypertension and diabetes, and family history of GC. BMI was calculated as weight in kg that was divided by height's square in meters. Family history of GC referred to at least one of the relatives (father/mother, brother/sister, son/daughter, and grandparents) with GC.

### 2.2. Tumor Markers Detection

Preoperative serum tumor markers were measured within 2 weeks before gastrectomy. Serum CEA, CA19-9, CA24-2, and CA72-4 levels were determined using an automatic electrochemistry luminescence immunoassay system (ROCHE E170; Roche, Germany). The cut-off points for these tumor markers were according to clinical standards. Serum level of CEA with >5 *μ*g/L, CA19-9 with >27 U/mL, CA24-2 with >20 U/mL, and CA72-4 with >6.9 U/mL were considered elevated levels, respectively.

### 2.3. Follow-Up

Information for vital status of all enrolled patients was obtained by regular telephone follow-up once a year. Follow-up assessment included physical examination, electronic gastroduodenoscopy, dynamic computed tomography (CT) scan, and laboratory testing. The latest follow-up date was November 2020. Overall survival (OS) was defined as the time from surgery to the date of last follow-up, or the date of death, whichever came first.

### 2.4. Statistical Analysis

The statistical analysis was performed using SPSS 21.0 and R software packages. All tests were 2-sided, and *P* < 0.05 was considered statistically significant. Differences among the groups were analyzed using the Pearson's Chi-square test. Survival analysis was performed with the Kaplan–Meier method, and differences between survival curves were compared with a log-rank test. All variables with statistical significance in the univariate analysis were considered in a multivariate Cox proportional hazard model in order to identify the independent factors. The prognostic abilities of the prognostic factors and predictive models were compared by calculating the estimated area under the curve (AUC). For nomogram construction, the rms package and survival package in R software were used. Patients were randomly divided into modeling cohort and validation cohort, and the sample size of the modeling group accounts for 70% of the total GC cases. The performance of the nomogram was evaluated using a concordance index (C-index) and calibration plots with 1000 bootstrap resamples. There is no multicollinearity between independent variables through variance expansion factor analysis.

## 3. Results

### 3.1. Patient Characteristics

In total, 928 GC cases were included in this study, of whom 652 (70.3%) were male and 276 (29.7%) were female. The median age at diagnosis for participants was 60.0 (range 13.0–91.0) years. Based on the TNM staging system, 90 (10.7%), 312 (33.6%), and 517 (55.7%) of patients had stage I, II, and III disease, respectively. Elevated CEA, CA19-9, CA24-2, and CA72-4 levels were identified in 194 (20.91%), 231 (24.89%), 154 (16.59%), and 261 (28.13%) of the participants, respectively. Patients were further divided into five groups according to the number of positive tumor markers (NPTM). Among all the case cohort, patients with four-negative or four-positive tumor markers accounted for 49.5% and 4.2%, respectively, and the proportions of patients with one-, two-, and three-positive tumor markers were 26.3%, 12.7%, and 7.3%, respectively. Postoperative chemotherapy was performed in 662 (71.3%) patients. The median time of follow-up was 33.78 months. The OS rate was 54.20% (Table 1).

### 3.2. Association between NPTM and Clinic-Pathological Traits

Table 2 and Table S1 show the relationship between NPTM as well as four preoperative serum tumor makers and clinic-pathological traits. Compared with patients younger than 60 years old, patients older than 60 years tended to have more positive tumor markers (*P* = 0.004). Poorly differentiated patients accounted for a large proportion in each NPTM subgroup, and patients tended to have intermediate or poor histological grades when all tumor markers were negative (*P* = 0.014). In addition, NPTM is positively proportional to tumor size and TNM stage, and the more NPTM, the greater the proportion of patients with tumors more than 5 cm in diameter (*P* < 0.001) and advanced patients (*P* < 0.001). Similar results were observed for the four preoperative serum tumor makers (CEA, CA19-9, CA24-2, and CA72-4) (Table S1).

### 3.3. Association between NPTM and GC Prognosis

**Figure 1:**
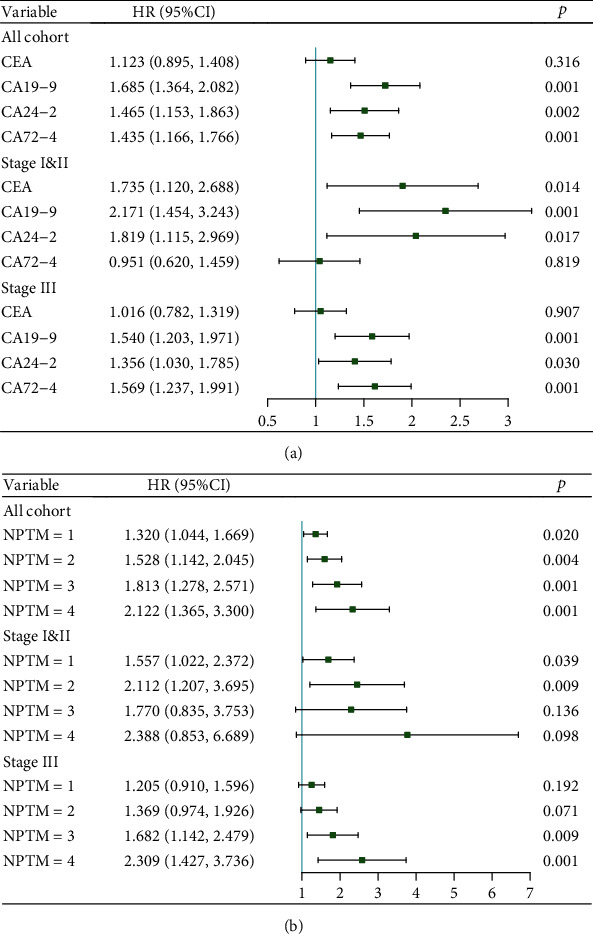
Multivariate Cox regression analysis for four tumor markers (a) and NPTM (b) with GC overall survival. Age, differentiation grade, tumor location, tumor size, and pTNM stage were adjusted in all cohort group; diabetes, differentiation grade, tumor location, and tumor size were adjusted in stage I&II group; tumor location and tumor size were adjusted in stage III group. NPTM = 0 was used as the reference group for Figure 1(b).

The Kaplan–Meier curves showed that with the increase of NPTM, GC patients in different stages all had remarkably poorer OS (all *P* < 0.01) (Figure S1). The 1-, 3-, and 5-year OS for GC patients with different positive tumor markers and NPTM were shown in Table S2, demonstrating that when NPTM > 1, the 1-, 3-, and 5-year survival rates of patients were lower than the survival rate of any single tumor marker positive, and with the increase of NPTM, the OS rate of GC patients gradually decreased. In all GC patients, univariate analysis showed that the elevation of all four tumor markers and NPTM was associated with decreased OS of GC. When stratified by stages, in earlier stage (Stage I&II), elevated CEA, CA19-9, and CA24-2 levels could predict poorer GC survival, while in stage III, elevated CA19-9, CA24-2, and CA72-4 were risk factors for OS. And, the increased NPTM remained as poor prognosis in each subgroup of stage (Table S3). As shown in Figure 1(a), the multivariable analysis revealed that CEA had independent and negative prognostic significance only in stage I&II subgroups, while elevated CA72-4 levels suggested a poor prognosis in all the case cohort, especially in more advanced patients; CA19-9 and CA24-2 had independently similar prognostic significance both in the case cohort or stratified by stage.  Figure 1(b) showed that NPTM had independent prognostic significance in all stratum patients, and with the increase of NPTM, the prognostic risk of GC patients increased.

**Figure 2:**
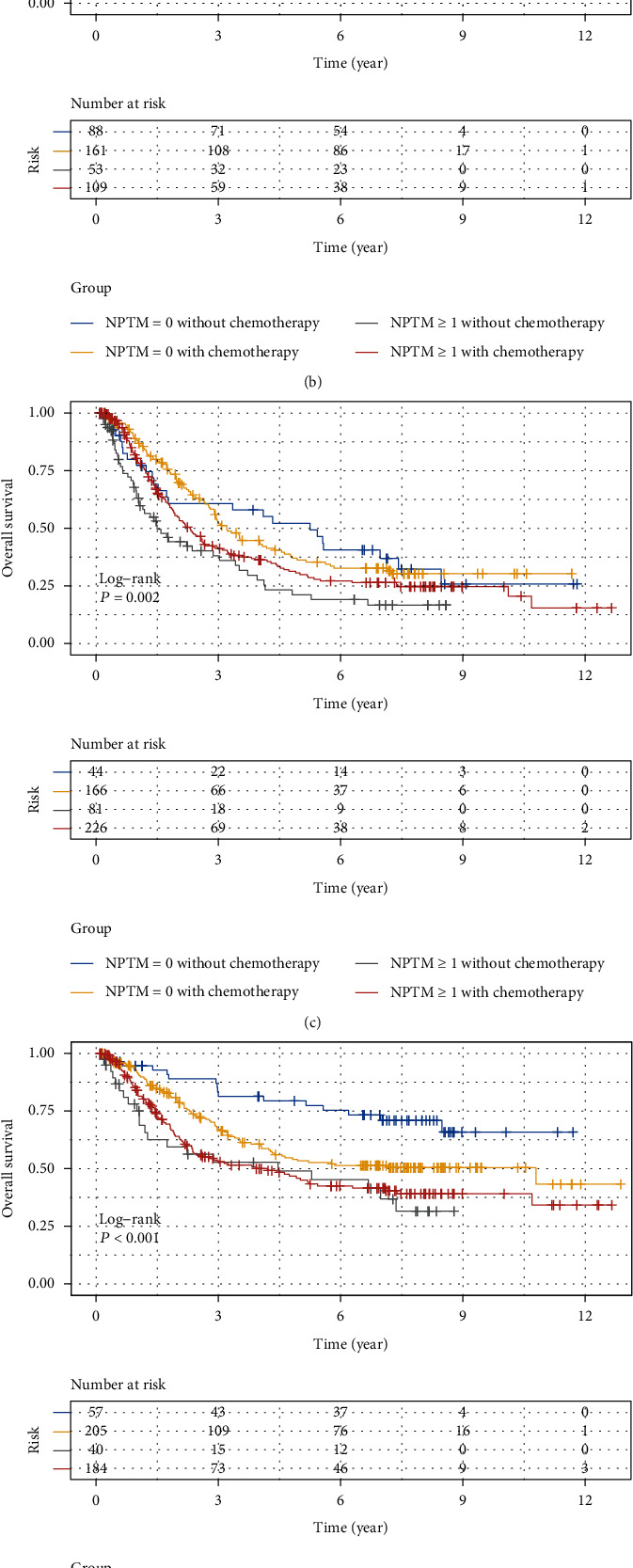
Kaplan–Meier curves for patients stratified by both NPTM and chemotherapy in all cohort (a), stage I&II cohort (b), stage III cohort (c), age ≤ 60 cohort (d), and age > 60 cohort (e).

Subsequently, when patients were assigned to NPTM = 0 and NPTM ≥ 1 subgroup, the survival disadvantage of the NPTM ≥ 1 group was more obvious, whether in the general population or stratified by stage, age, and whether undergoing postoperative chemotherapy (all *P* < 0.01) (Figure S2). Further, we explored the relationship between postoperative chemotherapy and prognosis in GC patients based on the NPTM (Figure 2 and Table S4). Patients with the all-negative tumor markers had a worse prognosis after postoperative chemotherapy in all cohort (*P* = 0.023, Figure 2(a)) or in age ≤ 60 subgroup (*P* = 0.012, Figure 2(d)); whereas in stage III, patients with positive tumor markers had a better prognosis after postoperative chemotherapy (*P* = 0.012, Figure 2(c)).

### 3.4. Prognostic Value of NPTM in Patients with GC

**Figure 3:**
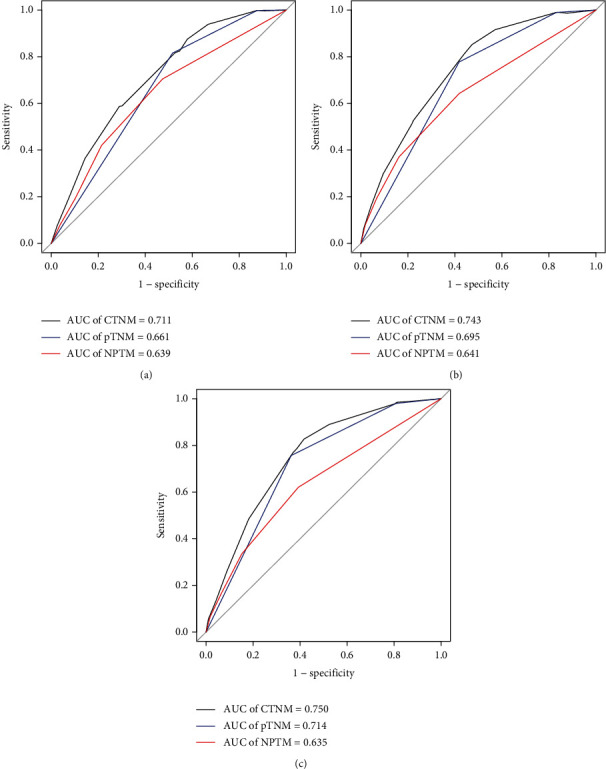
Comparison of the areas under the curves (AUC) for (a) 1-, (b) 3-, and (c) 5-year overall survival (OS) prediction. Abbreviations: AUC: area under the curve; NPTM: number of positive tumor markers; CTNM: the combination of NPTM and pTNM.

The AUC values were higher for NPTM than any individual tumor marker whether in all cohort or stratified by pTNM stage (Table S5). The receiver operating characteristic curves (ROC) of different groups are presented in Figure S3. Further by including preoperative NPTM into the conventional TNM staging system, we developed a novel prediction model of the combination of NPTM and TNM stage (CTNM) for GC patients. The AUC of the CTNM was significantly higher than that of the NPTM and TNM staging system (all *P* < 0.05) (Figure 3), and the 1-, 3-, and 5-year AUC values of the CTNM increased by 5%, 4.8%, and 3.6%, respectively, compared with TNM staging system.

### 3.5. The Nomogram for the Prediction of OS

**Figure 4:**
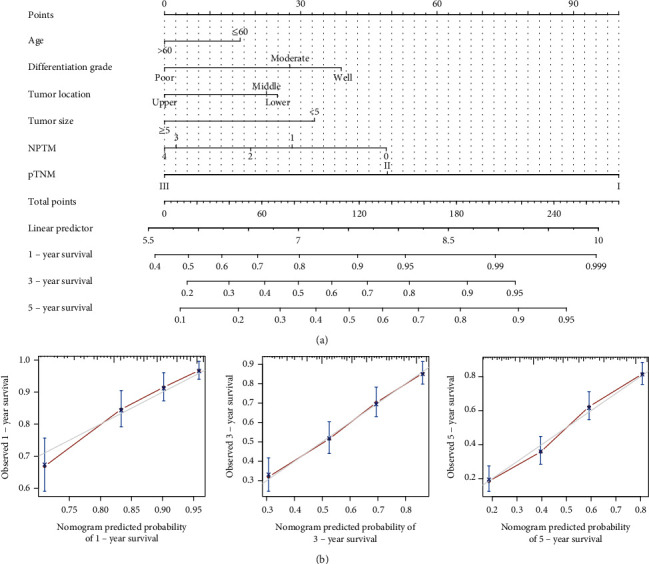
The nomogram constructed for the prognosis prediction of GC patients in modeling cohort. (a) Predictive nomogram for predicting 1-, 3-, and 5-year overall survival of GC patients. (b) The calibration curves of nomogram model predicting patients' 1-, 3-, and 5-year overall survival (OS). Nomogram model-predicted OS was plotted on the *x*-axis; actual OS was plotted on the *y*-axis. The reference line was 45 degree and indicated perfect calibration.

To better make individualized predictions of clinical outcomes, in the modeling cohort, we performed a prognostic nomogram to predict the 1-, 3-, and 5-year OS that integrated all independent prognostic factors including age, differentiation grade, tumor location, tumor size, NPTM, and pTNM stage in multivariable analysis (Table S6, Figure 4(a)). Calibration plots of the 1-, 3-, and 5-year OS nomogram based on bootstrap resampling validation showed the prediction (black line) was closely approximates the 45-degree line, which suggested the nomogram performing well with the ideal model (Figure 4(b)). The concordance indexes for nomogram (C − index = 0.706, 95% CI: 0.675–0.737) or CTNM model (C − index = 0.681, 95% CI: 0.656–0.706) were greater than those for the TNM staging system (C − index = 0.646, 95% CI: 0.624–0.668). The nomogram and calibration diagram based on validation cohort are shown in Figure S4, and the concordance index of the validation cohort was 0.709 (95% CI: 0.667–0.751).

## 4. Discussion

The traditional prognostic model of GC relies on the TNM staging system with a biological phenotype centered on tumor cells. In fact, even patients with the same TNM stage may have quite different prognosis. Serological tumor markers play an important role in the diagnosis, monitoring, prognosis, and even treatment of many cancers [18]. Preoperative tumor markers are not only easy to obtain but also noninvasive and low cost, as a tool widely used in clinical practice. In this study, we had proved the prognostic significance of NPTM, compared with individual tumor marker, presented better predictive ability. Incorporation of NPTM into TNM staging system could add more prognostic information to better identify patients with different outcomes.

In our study, analysis among all the clinicopathological variables showed that the number of positive tumor markers correlated with the age at diagnosis, tumor size, differentiation grade, and TNM stage as tumor markers produced by the tumor itself or by the normal tissue of the host in a response to tumor cells [19]. Therefore, patients with larger tumors and later TNM stages were more likely to experience increased NPTM. And studies have shown that age can affect the results of tumor marker measurements, and healthy older adults may even have higher levels of tumor markers than younger adults [20, 21]. However, the current conclusions on the relationship between tumor markers and the degree of differentiation of GC are not consistent. Several studies show that the positive rates of markers among the gastric cancer with different differentiation were not statistically significant, suggesting that the differentiation has little impact on gastric carcinoma marker level [22–25], while Jiexian et al. found that GC patients with poorer histological differentiation have higher positive rates of tumor markers [13]. Similar to the present study, there is also a study showing that the positive rate of CEA and CA72-4 is larger in patients with better differentiation [26]. Therefore, the relationship between NPTM and differentiation degree needs to be further studied.

After analyzing 41 studies that reported the relationship between CEA and the prognosis of GC, Deng et al. found that CEA is an independent risk factor for the prognosis of GC [27]. But in our study, this conclusion was verified only in earlier stage patients, and another study on early GC reached a consistent conclusion with us [28]. Abnormally elevated levels of CA19-9 was associated with poor prognosis in each stage, consistent with the conclusion of Kim et al. in a study of more than 1,200 gastric adenocarcinoma patients [29] and another two meta-analyses of 11,408 and 5072 GC patients, respectively [23, 30]. Similar with CA19-9, we found CA24-2 has independent prognostic significance in any stage of GC, and there are researches showed that the expression of CA24-2 was associated with the clinicopathologic characteristics of many kinds of gastrointestinal malignant tumors as well as in cancer prognosis [31–33]. In a Japanese study by Hamazoe et al. [34], the investigators concluded that CA 72-4 is highly specific to GC prognosis and could be a better tumor marker than CEA for GC patients. Our study also proved that CA72-4 played an important role in GC prognosis demonstrating an independent prognostic effect in all cohort and stage III patients, and the AUC value of CA72-4 is significantly higher than any other tumor markers. However, the prognostic effect of CA72-4 was not found in patients with stage I&II.

So far, a series of studies have explored the diagnostic and prognostic value of the combination of various serum tumor markers for cancers of digestive system [35], which may increase their utility in clinical practice. “CEA+CA19-9+CA24-2+CA72-4” combined detection has higher sensitivity and specificity in GC diagnosis [33]. The three markers CEA, CA19-9, and CA72-4 perform better when used in combination than used alone, whether in terms of GC staging before chemotherapy and surgery, or in improving sensitivity in diagnosis [36, 37]. Not only that, the combination of tumor markers also shows outstanding contributions in terms of prognosis. Toyoda et al. found that the combination of multiple tumor markers can improve the survival prediction of hepatocellular carcinoma compared with individual tumor marker [38]. The combined detection of preoperative serum CEA, CA19-9, and CA24-2 has independent prognostic value for the management of surgically treated colorectal cancer patients [39]. At present, there are only few studies exploring the contribution of tumor marker combinations to prognosis in GC [40] and found that the combined use of only two tumor markers (CEA and CA19-9) had significantly higher AUC values than CEA or CA19-9 alone for the prediction of 5-year OS. Here, we considered the combination of four common clinical tumor markers and constructed the NTPM model. The results showed that in all stages, the more positive preoperative serum tumor markers the patients had, the worse the prognosis. This may be due to the additive effects of tumor markers as prognostic factors. Multivariate analysis also showed that NPTM can be used as an independent predictor of GC prognosis no matter in all cohort or stratified by stage.

Interestingly, we did not find a significant association between postoperative chemotherapy and prognosis in patients with GC, However, when stratified according to the status of four tumor markers, it was found that postoperative chemotherapy did not benefit the survival for patients with all preoperative negative tumor markers, but for patients with more than one positive tumor marker in more advanced stage, postoperative chemotherapy could significantly improve the survival rate. The current studies have focused on the value of tumor markers in monitoring the curative effect of chemotherapy, and we have not found researches about the combined influence of preoperative tumor markers on chemotherapy treatment modalities. Our findings suggest that preoperative NPTM can determine whether patients need adjuvant chemotherapy and whether they can benefit from adjuvant chemotherapy, which may be helpful in planning treatment regimens.

The results of our analysis show that the predictive performance of NPTM was not only higher than any individual tumor marker but also presented greater prognostic accuracy when combination with TNM staging. Thus, NPTM can be used as an effective supplement to TNM staging. Finally, we constructed a nomogram based on the results of multivariate regression analysis, which could predict survival more precisely for resectable GC patients. The nomogram is an intuitive and widely used method to jointly diagnose or predict the onset or progression of diseases based on multiple valuable indicators [41].

However, our research also has some shortcomings. This was a single-center retrospective study, and the representativeness of the results was relatively limited. In addition, the preoperative level of a single tumor marker is easily affected by many factors. Elevated serum CEA level has been ascertained in various benign gastrointestinal and hepatic conditions such as pancreatitis, cholecystitis, and peptic ulcer disease [42, 43]; smoking and age may also affect the results of serum CEA measurement [20]. CA 19-9 levels can be very high in acute cholangitis, chronic hepatitis, and liver cirrhosis [44–46], and the use of drugs such as colchicine and *Ganoderma lucidum* spore powder (GLSP) may cause abnormal elevation of serum CA72-4 [47, 48]. In this study, we excluded some patients with serious complications and adjusted the patient's age, smoking, and other factors, but we could not adjust all the factors that may affect the level of tumor markers currently known. We also failed to stratify patients according to more detailed postoperative treatment methods and did not take radiotherapy and other adjuvant treatments into account. In short, multicenter, high-quality, stratified, and prospective studies are needed to further determine the clinical significance of combined serum tumor markers in the GC patients' prognosis.

## 5. Conclusions

In summary, we had evaluated the prognostic value of four tumor markers in patients with different stages of GC in a larger sample size, which can help us understand the value of these tumor markers more comprehensively, and the NPTM model we constructed showed better prognostic accuracy than each individual tumor markers and could be used as an effective supplement to TNM staging. Finally, the nomogram we constructed is a simple and cheap prognostic prediction tool that can be used in clinics.

## Figures and Tables

**Table 1:** Characteristics of 928 GC patients.

Variables	*N* of patients	%	Variables	*N* of patients	%
Gender			pTNM		
Female	276	29.7	I	99	10.7
Male	652	70.3	II	312	33.6
Age			III	517	55.7
≤60	486	52.4	CEA		
>60	442	47.6	Normal	734	79.1
Body mass index			Elevated	194	20.9
<24	546	58.8	CA19-9		
≥ 24	382	41.2	Normal	697	75.1
Hypertension			Elevated	231	24.9
No	750	80.8	CA24-2		
Yes	178	19.2	Normal	774	83.4
Diabetes			Elevated	154	16.6
No	846	91.2	CA72-4		
Yes	82	8.8	Normal	667	71.9
Family history			Elevated	261	28.1
No	799	86.1	NPTM		
Yes	129	13.9	0	459	49.5
Differentiation grade			1	244	26.3
Well	204	22.0	2	118	12.7
Moderate	181	19.5	3	68	7.3
Poorly	543	58.5	4	39	4.2
Tumor location			Postoperative chemotherapy		
Upper	183	19.7	No	266	28.7
Middle	226	24.4	Yes	662	71.3
Lower	519	55.9	Overall survival		
Tumor size (cm)			Alive	503	54.2
<5	370	39.9	Death	425	45.8
≥ 5	558	60.1			

Abbreviations: GC: gastric cancer; BMI: body mass index; TNM: tumor-node-metastasis staging; CEA: carcinoembryonic antigen; CA: carbohydrate antigen; NPTM: number of positive tumor markers.

**Table 2:** Correlation between NPTM and major clinic-pathological traits.

Patient characteristics	NPTM					*P* value^∗^
	0	1	2	3	4	
Gender						
Female	150 (32.7)	65 (26.6)	30 (25.4)	18 (26.5)	13 (33.3)	0.324
Male	309 (67.3)	179 (73.4)	88 (74.6)	50 (73.5)	26 (66.7)	
Age at diagnosis						
≤60	262 (57.1)	130 (53.3)	53 (44.9)	27 (39.7)	14 (35.9)	**0.004**
>60	197 (42.9)	114 (46.7)	65 (55.1)	41 (60.3)	25 (64.1)	
Body mass index						
<24	260 (56.6)	146 (59.8)	71 (60.2)	43 (63.2)	26 (66.7)	0.622
≥ 24	199 (43.4)	98 (40.2)	47 (39.8)	25 (36.8)	13 (33.3)	
Family history						
No	397 (86.5)	202 (82.8)	103 (87.3)	62 (91.2)	35 (89.7)	0.363
Yes	62 (13.5)	42 (17.2)	15 (12.7)	6 (8.8)	4 (10.3)	
Hypertension						
No	375 (81.7)	201 (82.4)	96 (81.4)	47 (69.1)	31 (79.5)	0.154
Yes	84 (18.3)	43 (17.6)	22 (18.6)	21 (30.9)	8 (20.5)	
Diabetes						
No	425 (92.6)	222 (91.0)	101 (85.6)	64 (94.1)	34 (87.2)	0.125
Yes	34 (7.4)	22 (9.0)	17 (14.4)	4 (5.9)	5 (12.8)	
Differentiation grade						
Well	81 (17.6)	58 (23.8)	31 (26.3)	20 (29.4)	14 (35.9)	**0.014**
Moderate	97 (21.1)	42 (17.2)	24 (20.3)	16 (23.5)	2 (5.1)	
Poorly	281 (61.2)	144 (59.0)	63 (53.4)	32 (47.1)	23 (59.0)	
Tumor location						
Upper	75 (16.3)	53 (21.7)	27 (22.9)	18 (26.5)	10 (25.6)	0.395
Middle	118 (25.7)	56 (23.0)	28 (23.7)	17 (25.0)	7 (17.9)	
Lower	266 (58.0)	135 (55.3)	63 (53.4)	33 (48.5)	22 (56.4)	
Tumor size (cm)						
<5	256 (55.8)	115 (47.1)	48 (40.7)	21 (30.9)	12 (30.8)	**<0.001**
≥ 5	203 (44.2)	129 (52.9)	70 (59.3)	47 (69.1)	27 (69.2)	
pTNM						
I	74 (16.1)	19 (7.8)	5 (4.2)	1 (1.5)	0 (0.0)	**<0.001**
II	175 (38.1)	79 (32.4)	33 (28.0)	16 (23.5)	9 (23.1)	
III	210 (45.8)	146 (59.8)	80 (67.8)	51 (75.0)	30 (76.9)	
Postoperative chemotherapy						
No	132 (28.8)	61 (25.0)	37 (31.4)	21 (30.9)	15 (38.5)	0.404
Yes	327 (71.2)	183 (75.0)	81 (68.6)	47 (69.1)	24 (61.5)	

Abbreviations: NPTM: number of positive tumor markers; BMI: body mass index; TNM: tumor-node-metastasis staging. ^∗^Difference between groups was tested by Chi-square test. Statistically significant values are in bold.

## Data Availability

The data used to support the findings of this study are included within the article and the supplementary information file.

## Supplementary Materials

Supplementary Materials Table S1: correlations between four preoperative serum tumor markers and major clinic-pathological traits. Table S2: the 1-, 3-, and 5-year OS for GC patients with different positive tumor markers and NPTM. Table S3: univariate analysis of OS in the study cohort of GC. Table S4: pairwise comparison results of Figure 2. Table S5: comparisons of the AUC between individual tumor markers and the NPTM for OS in different GC patients. Table S6: univariate and multivariate analyses of survival in the modeling group. Figure S1: relationships among the NPTM groups and overall survival in all cohort (A), stage I&II cohort (B), and stage III cohort (C). NPTM: number of positive tumor markers. Figure S2: multivariate regression analysis for NPTM and GC overall survival in all cohort and different subgroups. Figure S3: ROC curves for predicting 1-, 3-, and 5-year OS of CEA, CA19-9, CA24-2, CA72-4, and NPTM in all cohort (A, B, and C), in stage I&II cohort (D, E, and F), and in stage III cohort (G, H, and I). Figure S4: the nomogram constructed for the prognosis prediction of GC patients in validation cohort. (A) Predictive nomogram for predicting 1-, 3-, and 5-year overall survival of GC patients. (B) The calibration curves of nomogram model predicting patients' 1-, 3-, and 5-year overall survival (OS).
