# Supplementary material for: The Number of Positive Tumor Markers (NPTM) Achieves Higher Value in the Prognosis Prediction of Gastric Cancer
Source: Dis Markers. 2022 Nov 28;2022:5145918. doi: 10.1155/2022/5145918 (PMC9722318; doi:10.1155/2022/5145918)
Supplement: Supplementary Materials — Table S1: correlations between four preoperative serum tumor markers and major clinic-pathological traits. Table S2: the 1-, 3-, and 5-year OS for GC patients with different positive tumor markers and NPTM. Table S3: univariate analysis of OS in the study cohort of GC. Table S4: pairwise comparison results of Figure 2. Table S5: comparisons of the AUC between individual tumor markers and the NPTM for OS in different GC patients. Table S6: univariate and multivariate analyses of survival in the modeling group. Figure S1: relationships among the NPTM groups and overall survival in all cohort (A), stage I&II cohort (B), and stage III cohort (C). NPTM: number of positive tumor markers. Figure S2: multivariate regression analysis for NPTM and GC overall survival in all cohort and different subgroups. Figure S3: ROC curves for predicting 1-, 3-, and 5-year OS of CEA, CA19-9, CA24-2, CA72-4, and NPTM in all cohort (A, B, and C), in stage I&II cohort (D, E, and F), and in stage III cohort (G, H, and I). Figure S4: the nomogram constructed for the prognosis prediction of GC patients in validation cohort. (A) Predictive nomogram for predicting 1-, 3-, and 5-year overall survival of GC patients. (B) The calibration curves of nomogram model predicting patients' 1-, 3-, and 5-year overall survival (OS). [file 5145918.f1.docx]

**Supplemental Digital Content**

**Table S1: Correlations between four preoperative serum tumor markers and major clinic-pathological traits.**

**Table S2. The 1-, 3-, and 5-year OS for GC patients with different positive tumor markers and NPTM.**

**Table S3. Univariate analysis of OS in the study cohort of GC.**

**Table S4.** **Pairwise comparison results of Figure 2.**

**Table S5: Comparisons of the AUC between individual tumor markers and the NPTM for OS in different GC patients.**

**TABLE S6: Univariate and multivariate analysis of survival in the modeling group.**

**Figure S1. Relationships among the NPTM groups and overall survival in all cohort (A), stage I&II cohort (B) and stage III cohort (C). NPTM, number of positive tumor markers.**

**Figure S2. Multivariate regression analysis for NPTM and GC overall survival in all cohort and different subgroups.**

**Figure S3. ROC curves for predicting 1-, 3-, and 5-year OS of CEA, CA19-9, CA24-2, CA72-4 and NPTM in all cohort (A, B, C); in stage I&II cohort (D, E, F) and in stage III cohort (G, H, I).**

**Figure S4. The nomogram constructed for the prognosis prediction of GC patients in validation cohort. (A) Predictive nomogram for predicting 1-, 3- and 5-year overall survival of GC patients. (B) The calibration curves of nomogram model predicting patients’ 1-, 3-, and 5-year overall survival (OS).**

**Table S1: Correlation between four preoperative serum tumor makers and major clinic-pathological traits.**

| Patient characteristics | CEA (+) | CEA (-) | *P* value* | CA19-9 (+) | CA19-9 (-) | *P* value* | CA24-2 (+) | CA24-2 (-) | *P* value* | CA72-4 (+) | CA72-4 (-) | *P* value* |
| --- | --- | --- | --- | --- | --- | --- | --- | --- | --- | --- | --- | --- |
| Gender |  |  |  |  |  |  |  |  |  |  |  |  |
| Female | 42 (21.6) | 234 (31.9) | **0.006** | 67 (29.0) | 209 (30.0) | 0.777 | 46 (29.9) | 230 (29.7) | 0.969 | 76 (29.1) | 200 (30.0) | 0.795 |
| Male | 152 (78.4) | 500 (68.1) |  | 164 (71.0) | 488 (70.0) |  | 108 (70.1) | 544 (70.3) |  | 185 (70.9) | 467 (70.0) |  |
| Age at diagnosis |  |  |  |  |  |  |  |  |  |  |  |  |
| ≤60 | 82 (42.3) | 404 (55.0) | **0.002** | 94 (40.7) | 392 (56.2) | **<0.001** | 63 (40.9) | 423 (54.7) | **0.002** | 134 (51.3) | 352 (52.8) | 0.694 |
| ＞60 | 112 (57.7) | 330 (45.0) |  | 137 (59.3) | 305 (43.8) |  | 91 (59.1) | 351 (45.3) |  | 127 (48.7) | 315 (47.2) |  |
| Body mass index |  |  |  |  |  |  |  |  |  |  |  |  |
| ＜24 | 119 (61.3) | 427 (58.2) | 0.426 | 143 (61.9) | 403 (57.8) | 0.274 | 90 (58.4) | 456 (58.9) | 0.913 | 169 (64.8) | 377 (56.5) | **0.022** |
| ≥24 | 75 (38.7) | 307 (41.8) |  | 88 (38.1) | 294 (42.2) |  | 64 (41.6) | 318 (41.1) |  | 92 (35.2) | 290 (43.5) |  |
| Family history |  |  |  |  |  |  |  |  |  |  |  |  |
| No | 164 (84.5) | 635 (86.5) | 0.479 | 209 (90.5) | 590 (84.6) | **0.026** | 138 (89.6) | 661 (85.4) | 0.168 | 223 (85.4) | 576 (86.4) | 0.717 |
| Yes | 30 (15.5) | 99 (13.5) |  | 22 (9.5) | 107 (15.4) |  | 16 (10.4) | 113 (14.6) |  | 38 (14.6) | 91 (13.6) |  |
| Hypertension |  |  |  |  |  |  |  |  |  |  |  |  |
| No | 157 (80.9) | 593 (80.8) | 0.965 | 178 (77.1) | 572 (82.1) | 0.094 | 110 (71.4) | 640 (82.7) | **0.001** | 213 (81.6) | 537 (80.5) | 0.702 |
| Yes | 37 (19.1) | 141 (19.2) |  | 53 (22.9) | 125 (17.9) |  | 44 (28.6) | 134 (17.3) |  | 48 (18.4) | 130 (19.5) |  |
| Diabetes |  |  |  |  |  |  |  |  |  |  |  |  |
| No | 170 (87.6) | 676 (92.1) | 0.051 | 206 (89.2) | 640 (91.8) | 0.220 | 137 (89.0) | 709 (91.6) | 0.292 | 239 (91.6) | 607 (91.0) | 0.785 |
| Yes | 24 (12.4) | 58 (7.9) |  | 25 (10.8) | 57 (8.2) |  | 17 (11.0) | 65 (8.4) |  | 22 (8.4) | 60 (9.0) |  |
| Differentiation grade |  |  |  |  |  |  |  |  |  |  |  |  |
| Well | 56 (28.9) | 148 (20.2) | **0.026** | 60 (26.0) | 144 (20.7) | 0.226 | 48 (31.2) | 156 (20.2) | **0.011** | 72 (27.6) | 132 (19.8) | **0.018** |
| Moderate | 38 (19.6) | 143 (19.5) |  | 41 (17.7) | 140 (20.1) |  | 26 (16.9) | 155 (20.0) |  | 41 (15.7) | 140 (21.0) |  |
| Poorly | 100 (51.5) | 443 (60.4) |  | 130 (56.3) | 413 (59.3) |  | 80 (51.9) | 463 (59.8) |  | 148 (56.7) | 395 (59.2) |  |
| Tumor location |  |  |  |  |  |  |  |  |  |  |  |  |
| Upper | 57 (29.4) | 126 (17.2) | **0.001** | 56 (24.2) | 127 (18.2) | 0.088 | 36 (23.4) | 147 (19.0) | 0.329 | 52 (19.9) | 131 (19.6) | 0.714 |
| Middle | 43 (22.2) | 183 (24.9) |  | 48 (20.8) | 178 (25.5) |  | 32 (20.8) | 194 (25.1) |  | 68 (26.1) | 158 (23.7) |  |
| Lower | 94 (48.5) | 425 (57.9) |  | 127 (55.0) | 392 (56.2) |  | 86 (55.8) | 433 (55.9) |  | 141 (54.0) | 378 (56.7) |  |
| Tumor size (cm) |  |  |  |  |  |  |  |  |  |  |  |  |
| ＜5 | 79 (40.7) | 373 (50.8) | **0.012** | 96 (41.6) | 356 (51.1) | **0.012** | 50 (32.5) | 402 (51.9) | **<0.001** | 97 (37.2) | 355 (53.2) | **<0.001** |
| ≥5 | 115 (59.3) | 361 (49.2) |  | 135 (58.4) | 341 (48.9) |  | 104 (67.5) | 372 (48.1) |  | 164 (62.8) | 312 (46.8) |  |
| pTNM |  |  |  |  |  |  |  |  |  |  |  |  |
| I | 6 (3.1) | 93 (12.7) | **<0.001** | 9 (3.9) | 90 (12.9) | **<0.001** | 2 (1.3) | 97 (12.5) | **<0.001** | 15 (5.7) | 84 (12.6) | **<0.001** |
| II | 54 (27.8) | 258 (35.1) |  | 64 (27.7) | 248 (35.6) |  | 42 (27.3) | 270 (34.9) |  | 69 (26.4) | 243 (36.4) |  |
| III | 134 (69.1) | 383 (52.2) |  | 158 (68.4) | 359 (51.5) |  | 110 (71.4) | 407 (52.6) |  | 177 (67.8) | 340 (51.0) |  |
| Postoperative chemotherapy |  |  |  |  |  |  |  |  |  |  |  |  |
| No | 51 (26.3) | 215 (29.3) | 0.411 | 77 (33.3) | 189 (27.1) | 0.07 | 56 (36.4) | 210 (27.1) | **0.021** | 74 (28.4) | 192 (28.8) | 0.896 |
| Yes | 143 (73.7) | 519 (70.7) |  | 154 (66.7) | 508 (72.9) |  | 98 (63.6) | 564 (72.9) |  | 187 (71.6) | 475 (71.2) |  |

**Abbreviations:** BMI, body mass index; TNM, tumor-node-metastasis staging; CEA, carcinoembryonic antigen; CA, carbohydrate antigen.

*Difference between groups was tested by Chi-square test.

Statistically significant values are in bold.

**Table S2. The 1-, 3-, and 5-year OS for GC patients with different positive tumor markers and NPTM.**

| Variables | 1-OS (%) | 3-OS (%) | 5-OS (%) |
| --- | --- | --- | --- |
| CEA | 87.0 | 64.8 | 54.2 |
| CA19-9 | 89.2 | 67.0 | 56.6 |
| CA24-2 | 88.2 | 65.0 | 54.3 |
| CA72-4 | 89.4 | 66.2 | 54.6 |
| NPTM=1 | 85.1 | 59.3 | 46.4 |
| NPTM=2 | 75.7 | 46.1 | 34.6 |
| NPTM=3 | 78.1 | 39.9 | 32.3 |
| NPTM=4 | 75.0 | 27.1 | 21.7 |

**Abbreviations:** OS, overall survival; CEA, carcinoembryonic antigen; CA, carbohydrate antigen; NPTM, number of positive tumor markers.

**Table S3. Univariate analysis of OS in the study cohort of GC.**

| Risk factors | All cohort | |  | Stage I&II | |  | Stage III | |  |
| --- | --- | --- | --- | --- | --- | --- | --- | --- | --- |
|  | HR (95%CI) | *P* value |  | HR (95%CI) | *P* value |  | HR (95%CI) | *P* value |  |
| Gender |  |  |  |  |  |  |  |  |  |
| Female | 1 (ref) | 0.923 |  | 1 (ref) | 0.468 |  | 1 (ref) | 0.357 |  |
| Male | 1.010 (0.821-1.243) |  |  | 1.167 (0.769-1.769) |  |  | 1.120 (0.880-1.424) |  |  |
| Age |  |  |  |  |  |  |  |  |  |
| ≤60 | 1 (ref) | **0.049** |  | 1 (ref) | 0.177 |  | 1 (ref) | 0.074 |  |
| ＞60 | 1.210 (1.000-1.464) |  |  | 1.277 (0.896-1.819) |  |  | 1.229 (0.980-1.541) |  |  |
| Body mass index |  |  |  |  |  |  |  |  |  |
| ＜24 | 1 (ref) | 0.495 |  | 1 (ref) | 0.549 |  | 1 (ref) | 0.781 |  |
| ≥24 | 0.935 (0.770-1.134) |  |  | 0.896 (0.625-1.284) |  |  | 0.968 (0.769-1.218) |  |  |
| Family history |  |  |  |  |  |  |  |  |  |
| No | 1 (ref) | 0.437 |  | 1 (ref) | 0.601 |  | 1 (ref) | 0.415 |  |
| Yes | 0.892 (0.669-1.190) |  |  | 1.136 (0.703-1.837) |  |  | 0.860 (0.599-1.235) |  |  |
| Hypertension |  |  |  |  |  |  |  |  |  |
| No | 1 (ref) | 0.893 |  | 1 (ref) | 0.888 |  | 1 (ref) | 0.643 |  |
| Yes | 0.984 (0.772-1.253) |  |  | 0.967 (0.610-1.534) |  |  | 0.935 (0.703-1.243) |  |  |
| Diabetes |  |  |  |  |  |  |  |  |  |
| No | 1 (ref) | 0.060 |  | 1 (ref) | **0.044** |  | 1 (ref) | 0.677 |  |
| Yes | 1.335 (0.988-1.803) |  |  | 1.741 (1.014-2.989) |  |  | 1.080 (0.752-1.551) |  |  |
| Differentiation grade |  |  |  |  |  |  |  |  |  |
| Well | 1 (ref) | **0.008** |  | 1 (ref) | 0.129 |  | 1 (ref) | 0.104 |  |
| Moderate | 1.076 (0.794-1.459) | 0.635 |  | 0.794 (0.449-1.405) | 0.428 |  | 1.076 (0.747-1.548) | 0.695 |  |
| Poorly | 1.412 (1.100-1.812) | **0.007** |  | 1.284 (0.835-1.972) | 0.255 |  | 1.334 (0.980-1.816) | 0.067 |  |
| Tumor location |  |  |  |  |  |  |  |  |  |
| Upper | 1 (ref) | **<0.001** |  | 1 (ref) | **0.014** |  | 1 (ref) | **0.044** |  |
| Middle | 0.731 (0.557-0.960) | **0.024** |  | 0.666 (0.403-1.101) | 0.113 |  | 0.764 (0.552-1.056) | 0.103 |  |
| Lower | 0.621 (0.491-0.784) | **<0.001** |  | 0.528 (0.344-0.810) | **0.003** |  | 0.701 (0.531-0.927) | **0.013** |  |
| Tumor size (cm) |  |  |  |  |  |  |  |  |  |
| ＜5 | 1 (ref) | **<0.001** |  | 1 (ref) | **<0.001** |  | 1 (ref) | **<0.001** |  |
| ≥5 | 2.071 (1.702-2.521) |  |  | 2.323 (1.627-3.317) |  |  | 1.490 (1.176-1.888) |  |  |
| pTNM |  |  |  |  |  |  |  |  |  |
| I | 1 (ref) | **<0.001** |  | / | / |  | / | / |  |
| II | 2.514 (1.506-4.197) | **<0.001** |  | / | / |  | / | / |  |
| III | 6.513 (3.990-10.630) | **<0.001** |  | / | / |  | / | / |  |
| Postoperative chemotherapy |  |  |  |  |  |  |  |  |  |
| No | 1 (ref) | 0.253 |  | 1 (ref) | 0.324 |  | 1 (ref) | 0.110 |  |
| Yes | 1.134 (0.914-1.406) |  |  | 1.212 (0.828-1.774) |  |  | 0.808(0.622-1.050) |  |  |
| CEA |  |  |  |  |  |  |  |  |  |
| Normal | 1 (ref) | **<0.001** |  | 1 (ref) | **0.002** |  | 1 (ref) | 0.296 |  |
| Elevated | 1.583 (1.271-1.972) |  |  | 1.962 (1.272-3.026) |  |  | 1.146 (0.888-1.478) |  |  |
| CA19-9 |  |  |  |  |  |  |  |  |  |
| Normal | 1 (ref) | **<0.001** |  | 1 (ref) | **<0.001** |  | 1 (ref) | **<0.001** |  |
| Elevated | 2.042 (1.663-2.509) |  |  | 2.493 (1.676-3.707) |  |  | 1.543 (1.213-1.963) |  |  |
| CA24-2 |  |  |  |  |  |  |  |  |  |
| Normal | 1 (ref) | **<0.001** |  | 1 (ref) | **<0.001** |  | 1 (ref) | **0.026** |  |
| Elevated | 1.896 (1.501-2.394) |  |  | 2.493 (1.554-4.001) |  |  | 1.356 (1.036-1.774) |  |  |
| CA72-4 |  |  |  |  |  |  |  |  |  |
| Normal | 1 (ref) | **<0.001** |  | 1 (ref) | 0.485 |  | 1 (ref) | **<0.001** |  |
| Elevated | 1.598 (1.303-1.960) |  |  | 1.162 (0.762-1.773) |  |  | 1.610 (1.271-2.039) |  |  |
| NPTM |  |  |  |  |  |  |  |  |  |
| 0 | 1 (ref) | **<0.001** |  | 1(ref) | **0.001** |  | 1(ref) | **0.001** |  |
| 1 | 1.565 (1.240-1.974) | **<0.001** |  | 1.699 (1.118-2.581) | **0.013** |  | 1.256 (0.950-1.661) | 0.110 |  |
| 2 | 2.096 (1.573-2.793) | **<0.001** |  | 2.560 (1.489-4.404) | **0.001** |  | 1.458 (1.039-2.047) | **0.029** |  |
| 3 | 2.367 (1.684-3.327) | **<0.001** |  | 2.593 (1.237-5.434) | **0.012** |  | 1.621 (1.101-2.386) | **0.014** |  |
| 4 | 3.378 (2.199-5.190) | **<0.001** |  | 3.194 (1.158-8.813) | **0.025** |  | 2.441 (1.513-3.938) | **<0.001** |  |

**Abbreviations:** HR, hazard ratio; CI, confidence interval; CEA, carcinoembryonic antigen; CA, carbohydrate antigen; TNM, tumor-node-metastasis staging; NPTM, number of positive tumor markers.

Statistically significant values are in bold.

**Table S4. Pairwise comparison result of Figure 3.**

| Group | Comparison | *χ2* | *P* |
| --- | --- | --- | --- |
| All cohort | “NPTM=0 without chemotherapy” vs “NPTM=0 with chemotherapy” | 5.190 | **0.023** |
|  | "NPTM=0 without chemotherapy" vs "NPTM≥1 without chemotherapy" | 26.775 | **＜0.001** |
|  | "NPTM=0 without chemotherapy" vs "NPTM≥1 with chemotherapy" | 26.958 | **＜0.001** |
|  | “NPTM=0 with chemotherapy” vs "NPTM≥1 without chemotherapy" | 17.573 | **＜0.001** |
|  | “NPTM=0 with chemotherapy” vs "NPTM≥1 with chemotherapy" | 19.557 | **＜0.001** |
|  | "NPTM≥1 without chemotherapy" vs "NPTM≥1 with chemotherapy" | 0.994 | 0.319 |
| stage I&II | “NPTM=0 without chemotherapy” vs “NPTM=0 with chemotherapy” | 1.384 | 0.239 |
|  | "NPTM=0 without chemotherapy" vs "NPTM≥1 without chemotherapy" | 9.627 | **0.002** |
|  | "NPTM=0 without chemotherapy" vs "NPTM≥1 with chemotherapy" | 11.296 | **0.001** |
|  | “NPTM=0 with chemotherapy” vs "NPTM≥1 without chemotherapy" | 5.045 | **0.025** |
|  | “NPTM=0 with chemotherapy” vs "NPTM≥1 with chemotherapy" | 7.027 | **0.008** |
|  | "NPTM≥1 without chemotherapy" vs "NPTM≥1 with chemotherapy" | 0.009 | 0.926 |
| stage III | “NPTM=0 without chemotherapy” vs “NPTM=0 with chemotherapy” | 0.031 | 0.859 |
|  | "NPTM=0 without chemotherapy" vs "NPTM≥1 without chemotherapy" | 5.610 | **0.018** |
|  | "NPTM=0 without chemotherapy" vs "NPTM≥1 with chemotherapy" | 1.562 | 0.211 |
|  | “NPTM=0 with chemotherapy” vs "NPTM≥1 without chemotherapy" | 12.617 | **＜0.001** |
|  | “NPTM=0 with chemotherapy” vs "NPTM≥1 with chemotherapy" | 4.536 | **0.033** |
|  | "NPTM≥1 without chemotherapy" vs "NPTM≥1 with chemotherapy" | 4.741 | **0.029** |
| Age≤60 | “NPTM=0 without chemotherapy” vs “NPTM=0 with chemotherapy” | 6.340 | **0.012** |
|  | "NPTM=0 without chemotherapy" vs "NPTM≥1 without chemotherapy" | 13.646 | **＜0.001** |
|  | "NPTM=0 without chemotherapy" vs "NPTM≥1 with chemotherapy" | 14.777 | **＜0.001** |
|  | “NPTM=0 with chemotherapy” vs "NPTM≥1 without chemotherapy" | 4.731 | **0.030** |
|  | “NPTM=0 with chemotherapy” vs "NPTM≥1 with chemotherapy" | 5.932 | **0.015** |
|  | "NPTM≥1 without chemotherapy" vs "NPTM≥1 with chemotherapy" | 0.521 | 0.470 |
| Age＞60 | “NPTM=0 without chemotherapy” vs “NPTM=0 with chemotherapy” | 0.494 | 0.482 |
|  | "NPTM=0 without chemotherapy" vs "NPTM≥1 without chemotherapy" | 12.063 | **0.001** |
|  | "NPTM=0 without chemotherapy" vs "NPTM≥1 with chemotherapy" | 13.680 | **＜0.001** |
|  | “NPTM=0 with chemotherapy” vs "NPTM≥1 without chemotherapy" | 11.134 | **0.001** |
|  | “NPTM=0 with chemotherapy” vs "NPTM≥1 with chemotherapy" | 14.683 | **＜0.001** |
|  | "NPTM≥1 without chemotherapy" vs "NPTM≥1 with chemotherapy" | 0.071 | 0.790 |

**Abbreviations:** *χ2*, chi-square test; NPTM, number of positive tumor markers.

Statistically significant values are in bold.

**TABLE S5: Comparison of the AUC between individual tumor markers and NPTM for OS in different GC patients.**

| Variables | AUC of all cohorts | | |  | AUC of stage I&II | | |  | AUC of stage III | | |  |
| --- | --- | --- | --- | --- | --- | --- | --- | --- | --- | --- | --- | --- |
|  | 1-year | 3-year | 5-year |  | 1-year | 3-year | 5-year |  | 1-year | 3-year | 5-year |  |
| CEA | 0.525 | 0.562 | 0.561 |  | 0.560 | 0.575 | 0.563 |  | 0.492 | 0.529 | 0.523 |  |
| CA19-9 | 0.594 | 0.596 | 0.595 |  | 0.612 | 0.614 | 0.605 |  | 0.567 | 0.562 | 0.563 |  |
| CA24-2 | 0.566 | 0.568 | 0.563 |  | 0.559 | 0.576 | 0.580 |  | 0.549 | 0.542 | 0.523 |  |
| CA72-4 | 0.598 | 0.580 | 0.559 |  | 0.458 | 0.526 | 0.523 |  | 0.623 | 0.592 | 0.563 |  |
| NPTM | 0.639 | 0.641 | 0.635 |  | 0.630 | 0.639 | 0.634 |  | 0.606 | 0.599 | 0.579 |  |

**Abbreviations:** CEA, carcinoembryonic antigen; CA, carbohydrate antigen; NPTM, number of positive tumor markers; AUC, area under the curve.

**TABLE S6: Univariate and multivariate analysis of survival in the modeling group.**

| Risk factors | N of patients | Univariate analysis | |  | Multivariate analysis | |  |
| --- | --- | --- | --- | --- | --- | --- | --- |
|  |  | HR (95%CI) | *P* value |  | HR (95%CI) | *P* value |  |
| Gender |  |  |  |  |  |  |  |
| Female | 186 | 1 (ref) | 0.612 |  | / | / |  |
| Male | 464 | 0.938 (0.733-1.201) |  |  | / |  |  |
| Age |  |  |  |  |  |  |  |
| ≤60 | 346 | 1 (ref) | **0.021** |  | 1 (ref) | **0.016** |  |
| ＞60 | 304 | 1.306 (1.041-1.639) |  |  | 1.334 (1.055-1.687) |  |  |
| Body mass index |  |  |  |  |  |  |  |
| ＜24 | 392 | 1 (ref) | 0.831 |  | / | / |  |
| ≥24 | 258 | 1.026 (0.814-1.292) |  |  | / |  |  |
| Family history |  |  |  |  |  |  |  |
| No | 566 | 1 (ref) | 0.756 |  | / |  |  |
| Yes | 84 | 0.946 (0.664-1.346) |  |  | / |  |  |
| Hypertension |  |  |  |  |  |  |  |
| No | 531 | 1 (ref) | 0.389 |  | / | / |  |
| Yes | 119 | 0.875 (0.646-1.186) |  |  | / |  |  |
| Diabetes |  |  |  |  |  |  |  |
| No | 595 | 1 (ref) | **0.042** |  | 1 (ref) | 0.463 |  |
| Yes | 55 | 1.455 (1.013-2.090) |  |  | 1.148 (0.794-1.659) |  |  |
| Differentiation grade |  |  |  |  |  |  |  |
| Well | 144 | 1 (ref) | **0.029** |  | 1 (ref) | **0.001** |  |
| Moderate | 127 | 1.058(0.736-1.521) | 0.762 |  | 1.204 (0.833-1.740) | 0.323 |  |
| Poorly | 379 | 1.409 (1.046-1.899) | **0.024** |  | 1.734 (1.276-2.356) | **<0.001** |  |
| Tumor location |  |  |  |  |  |  |  |
| Upper | 133 | 1 (ref) | **0.009** |  | 1 (ref) | **0.047** |  |
| Middle | 152 | 0.854 (0.618-1.181) | 0.340 |  | 0.724 (0.519-1.009) | 0.057 |  |
| Lower | 365 | 0.656 (0.495-0.870) | **0.003** |  | 0.703(0.527-0.937) | **0.016** |  |
| Tumor size (cm) |  |  |  |  |  |  |  |
| ＜5 | 322 | 1 (ref) | **<0.001** |  | 1 (ref) | **<0.001** |  |
| ≥5 | 328 | 2.101 (1.661-2.656) |  |  | 1.681 (1.320-2.141) |  |  |
| pTNM |  |  |  |  |  |  |  |
| I | 66 | 1 (ref) | **<0.001** |  | 1 (ref) | **<0.001** |  |
| II | 214 | 2.388 (1.263-4.515) | **0.007** |  | 2.186 (1.152-4.147) | **0.017** |  |
| III | 370 | 6.518 (3.552-11.961) | **<0.001** |  | 5.201 (2.813-9.616) | **<0.001** |  |
| Postoperative chemotherapy |  |  |  |  |  |  |  |
| No | 185 | 1 (ref) | 0.603 |  | / | / |  |
| Yes | 465 | 1.071 (0.827-1.386) |  |  | / |  |  |
| NPTM |  |  |  |  |  |  |  |
| 0 | 326 | 1 (ref) | **<0.001** |  | 1(ref) | **0.001** |  |
| 1 | 172 | 1.557 (1.182-2.053) | **0.002** |  | 1.345 (1.017-1.777) | **0.038** |  |
| 2 | 78 | 2.010 (1.421-2.845) | **<0.001** |  | 1.410 (0.989-2.010) | 0.057 |  |
| 3 | 49 | 2.583 (1.733-3.849) | **<0.001** |  | 2.101 (1.391-3.173) | **<0.001** |  |
| 4 | 25 | 3.204 (1.871-5.486) | **<0.001** |  | 2.069 (1.199-3.571) | **0.009** |  |


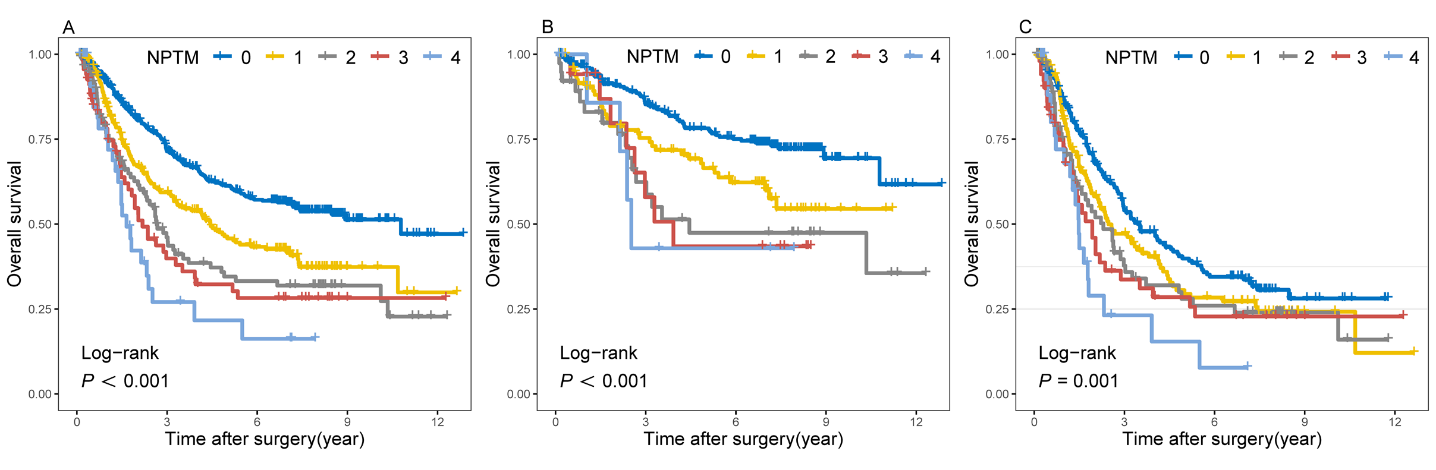


**Figure S1.** Relationships among the NPTM groups and overall survival in all cohort (A), stage I&II cohort (B) and stage III cohort (C). NPTM, number of positive tumor markers.


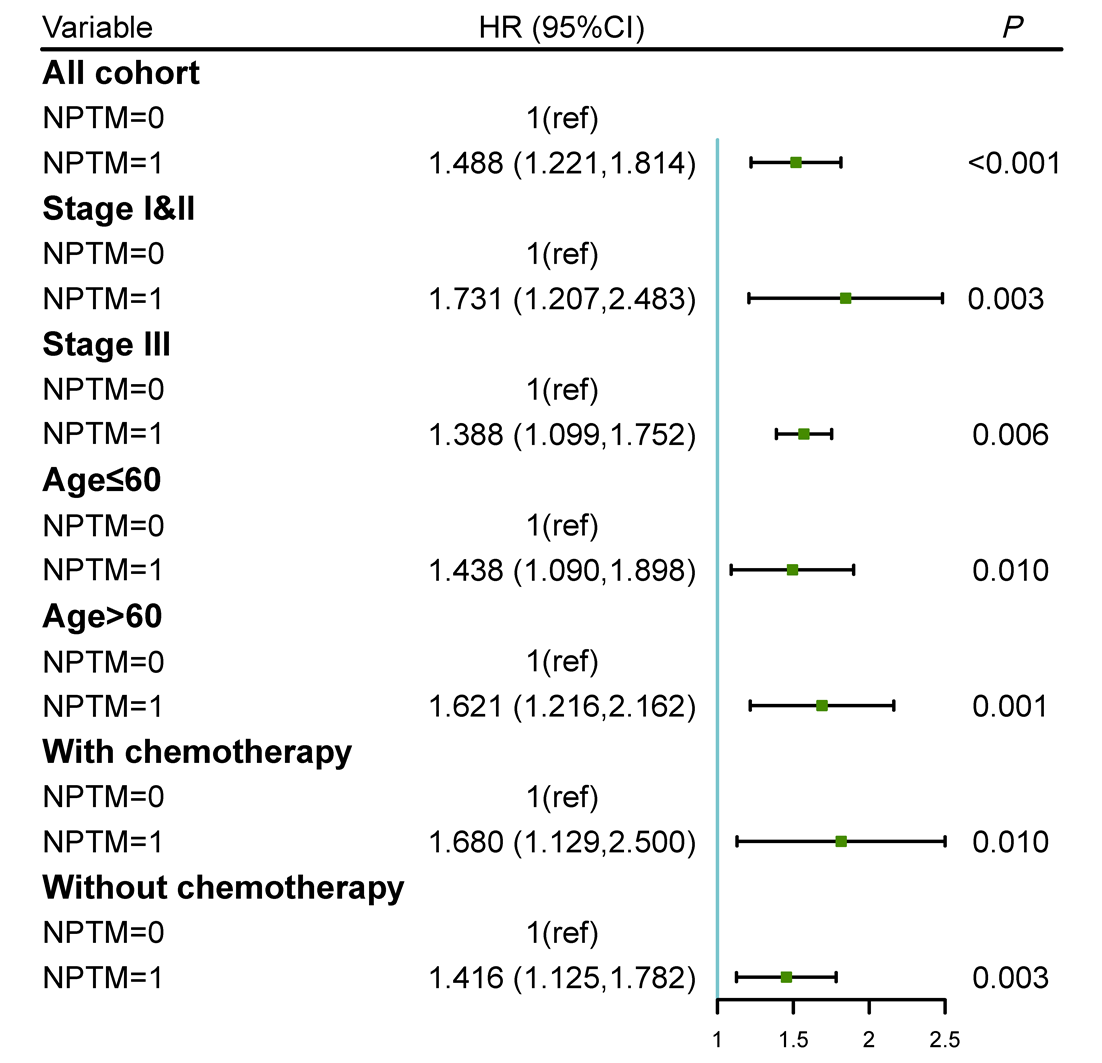


**Figure S2.** Multivariate regression analysis for NPTM and GC overall survival in all cohort and different subgroups.


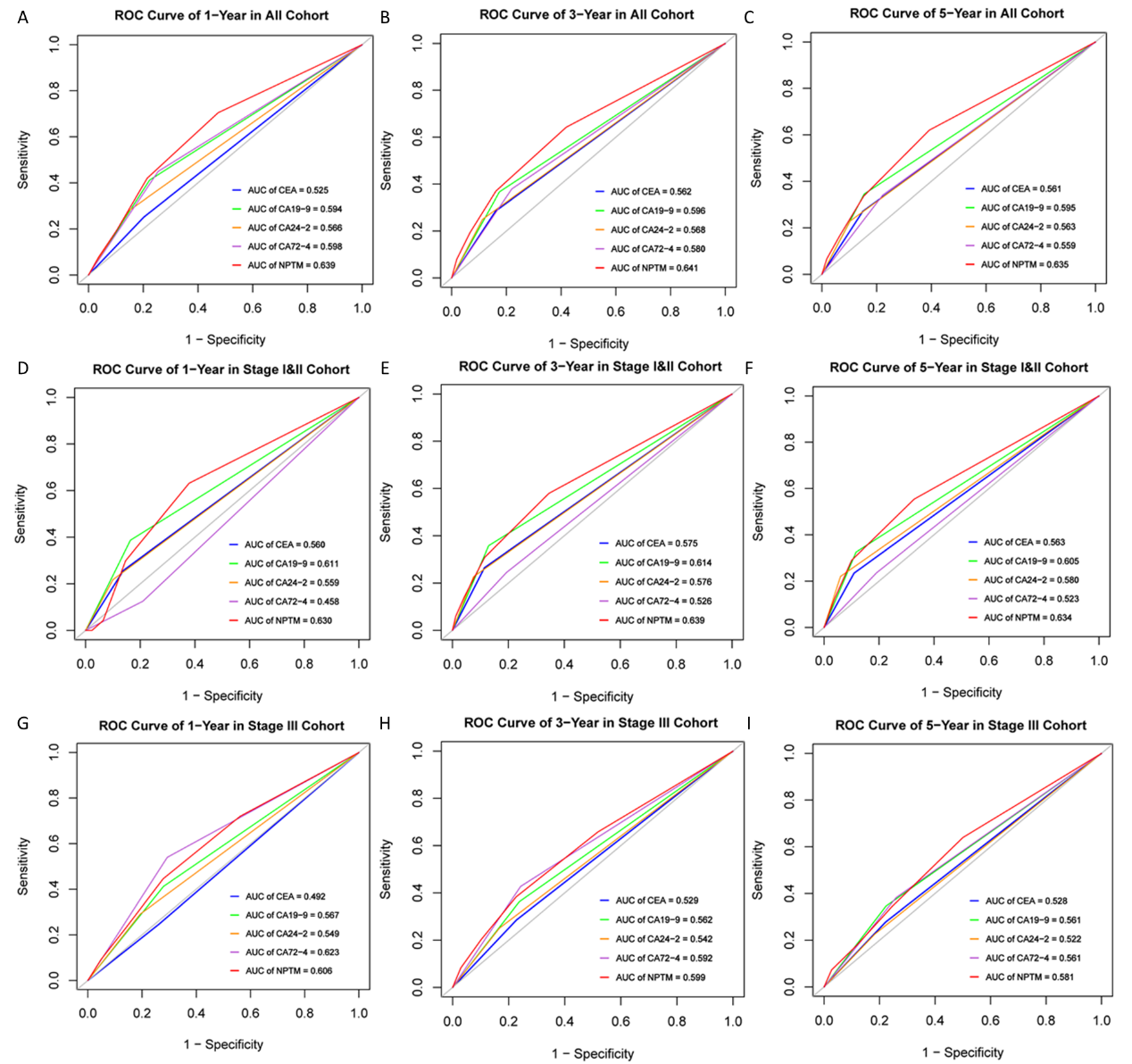


**Figure S3.** ROC curves for predicting 1-, 3-, and 5-year OS of CEA, CA19-9, CA24-2, CA72-4 and NPTM in all cohort (A, B, C); in stage I&II cohort (D, E, F) and in stage III cohort (G, H, I).


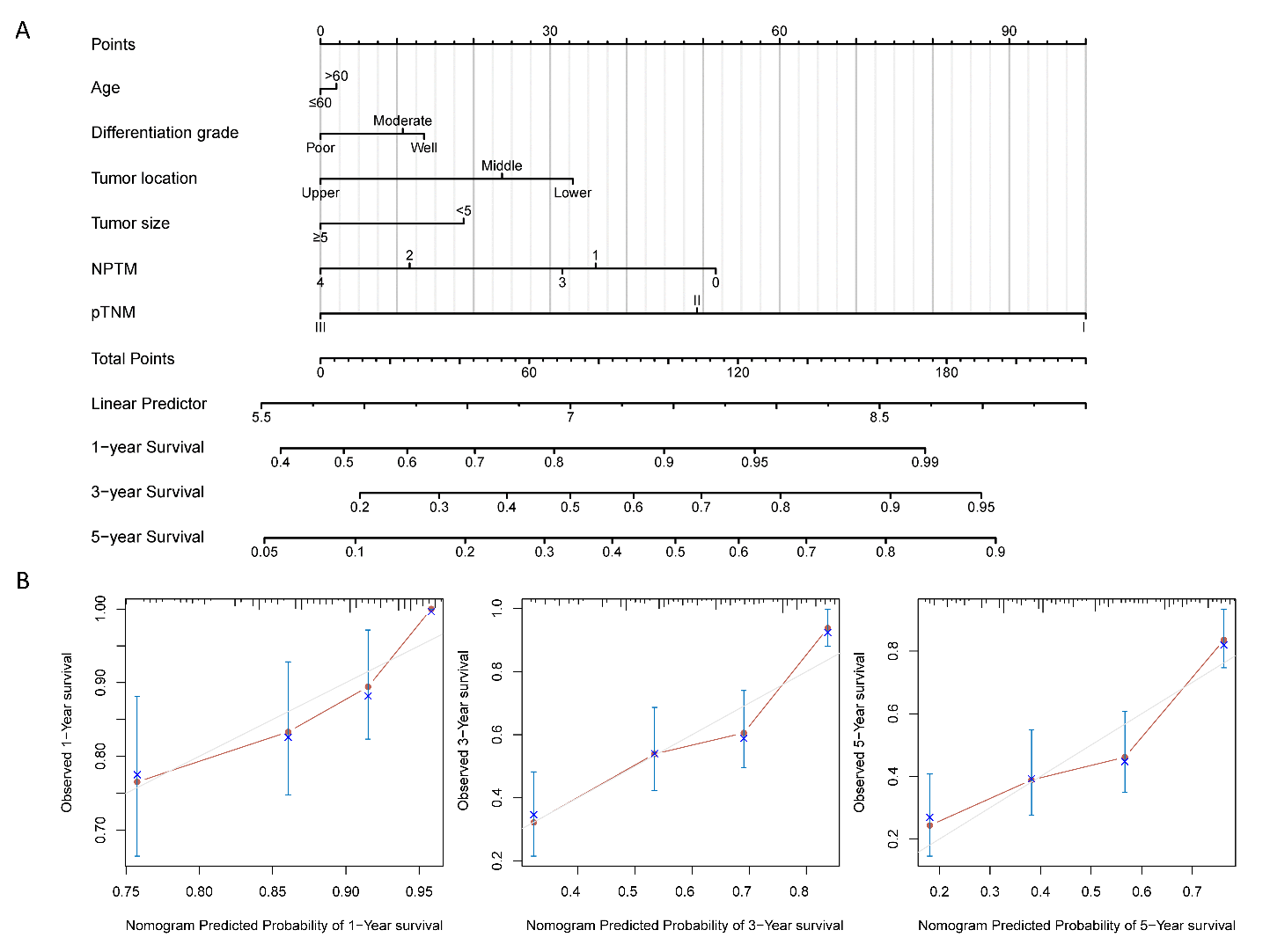


**Figure S4.** The nomogram constructed for the prognosis prediction of GC patients in validation cohort. **(**A) Predictive nomogram for predicting 1-, 3- and 5-year overall survival of GC patients. (B) The calibration curves of nomogram model predicting patients’ 1-, 3-, and 5-year overall survival (OS).
